# Effect of PRM1201 Combined With Adjuvant Chemotherapy on Preventing Recurrence and Metastasis of Stage III Colon Cancer: A Randomized, Double-Blind, Placebo-Controlled Clinical Trial

**DOI:** 10.3389/fonc.2021.618793

**Published:** 2021-03-03

**Authors:** Ru Jia, Ningning Liu, Guoxiang Cai, Yun Zhang, Haijuan Xiao, Lihong Zhou, Qing Ji, Ling Zhao, Puhua Zeng, Huaimin Liu, Jiege Huo, Xiaoqiang Yue, Yi Zhang, Chaojun Wu, Xiaoting Sun, Yuanyuan Feng, Hongjie Liu, Hui Liu, Zhifen Han, Youying Lai, Yanbo Zhang, Gang Han, Hangjun Gong, Yan Wang, Qi Li

**Affiliations:** ^1^ Department of Medical Oncology, Shuguang Hospital, Shanghai University of Traditional Chinese Medicine, Shanghai, China; ^2^ Academy of Integrative Medicine, Shanghai University of Traditional Chinese Medicine, Shanghai, China; ^3^ Department of Colorectal Cancer Surgery, Fudan University Shanghai Cancer Center, Shanghai, China; ^4^ Department of Gastrointestinal Surgery, Shuguang Hospital, Shanghai University of Traditional Chinese Medicine, Shanghai, China; ^5^ Department of Oncology, Hospital Affiliated to Shaanxi University of Chinese Medicine, Xianyang, China; ^6^ Department of Medical Oncology, Hunan University of Chinese Medicine Integrated Chinese and Western Medicine Affiliated Hospital, Changsha, China; ^7^ Department of Integrated Chinese and Western Medicine, Henan Cancer Hospital, Zhengzhou, China; ^8^ Department of Oncology, Affiliated Hospital of Integrated Traditional Chinese and Western Medicine, Nanjing University of Chinese Medicine, Nanjing, China; ^9^ Department of Traditional Chinese Medicine, Changzheng Hospital, Naval Medical University, Shanghai, China

**Keywords:** colon cancer, Chinese herbal medicine, cancer recurrence and metastasis, disease-free survival, clinical trial

## Abstract

**Background:**

Chemotherapy is the standard adjuvant treatment for colon cancer. Chinese herbal formula PRM1201 improves the efficacy of chemotherapy when used in combination with Cetuximab or Bevacizumab in patients with metastatic colorectal cancer. This study aims to explore the benefits of treatment with chemotherapy plus PRM1201 in the postoperative adjuvant setting.

**Methods:**

In this parallel-group study, patients who had undergone curative resection for stage III colon cancer were randomly assigned to receive adjuvant chemotherapy (FOLFOX q2w for 6 months, or CapeOx q3w for 6 months) plus PRM1201 (chemo+PRM1201 group) or adjuvant chemotherapy plus placebo (chemo+placebo group). The primary endpoint was disease-free survival (DFS), and the secondary endpoints were quality of life (QOL) and toxicity.

**Results:**

A total of 370 patients were randomly assigned to chemotherapy plus PRM1201 group (n = 184) and chemotherapy plus placebo group (n = 186). Up to October 30, 2019, 96 events of recurrence, metastasis, or death had been reported, of which 38 events were in the group of chemotherapy plus PRM1201 and 58 events in the chemo+placebo group. The 3-year DFS rate was 77.1 and 68.6% in the chemo+PRM1201 and chemo+placebo group, respectively (hazard ratio [HR], 0.63; 95% CI, 0.42 to 0.94). The QOL of patients in the chemo+PRM1201 group were significantly improved in terms of global quality of life, physical functioning, role functioning, emotional functioning, fatigue, and appetite loss. The incidence of grade 3 or 4 treatment-related adverse event (TRAEs) were similar between the two arms.

**Conclusions:**

Chemotherapy in combination with PRM1201 improved the adjuvant treatment of colon cancer. PRM1201 can be recommended as an effective option in clinical practice.

**Clinical Trial Registration:**

Chinese Clinical Trials Registry, identifier ChiCTR-IOR-16007719.

## Introduction

Colon cancer (CRC) is a common type of gastrointestinal tumor which has a high rate of fatality, ranking as the second leading cause of cancer-related death worldwide (1). After receiving standard treatments, such as surgery, and chemotherapy, recurrence and metastasis are still the most important factors related to the survival of patients (2). The International Duration Evaluation of Adjuvant Therapy (IDEA) study showed that more than 25% of patients with stage III colon cancer had recurrence or metastasis within 3 years after surgery even with standard adjuvant chemotherapy (3). Additionally, the 5-year survival rate for metastatic colon cancer is only 10% (4).

In addition to surgery and chemotherapeutic drugs, most Chinese patients seek help from traditional Chinese medicine during or after standard first-line therapy. As an important component of medicine in China nowadays, Chinese herbal medicine (CHM) plays an important role in cancer treatment and is covered by most Chinese health insurance. A large number of clinical trials have shown that CHM has its own advantages in coping with malignant tumors. The effects of CHM in alleviating symptoms, improving quality of life (QOL), and reducing side-effects and adverse events caused by chemotherapeutic drugs have been confirmed (5–7). However, few studies have focused on the effects of CHM treatment on survival outcomes in stage III colon cancer, and thus, its use remains controversial.

PRM1201 is a Chinese herbal formula composed of seven herbs. A network pharmacology study showed that the therapeutic targets of the active components of PRM1201 are closely related to colorectal cancer (8). Both *in vitro* and *in vivo* experiments showed that this formula can inhibit the invasion and metastasis of colon cancer (9, 10). A randomized, controlled, and double-blinded clinical trial confirmed that PRM1201 combined with chemotherapy and Cetuximab or Bevacizumab can prolong the progression free survival of metastatic colorectal cancer (11). To determine whether PRM1201 can benefit patients with colon cancer in an earlier stage, we conducted this prospective interventional study in patients with stage III colon cancer.

## Materials and Methods

### Study Design

This was a randomized, double-blinded, placebo-controlled clinical trial conducted in seven tertiary hospitals in China, including: Shuguang Hospital, Shanghai University of Traditional Chinese Medicine; Fudan University Shanghai Cancer Center; Hospital Affiliated to Shaanxi University of Chinese Medicine; Hunan University of Chinese Medicine Integrated Chinese and Western Medicine Affiliated Hospital; Henan Cancer Hospital; Affiliated Hospital of Integrated Traditional Chinese and Western Medicine, Nanjing University of Chinese Medicine; and Shanghai Changzheng Hospital.

The study protocol was in compliance with the Declaration of Helsinki and the “Good Clinical Practice” guidelines, and approved by the institutional research ethics committee of all medical centers. The study was registered in the Chinese Clinical Trial Registry (No.: ChiCTR-IOR-16007719). All patients provided signed informed consent before enrollment in the study.

### Inclusion and Exclusion Criteria

The inclusion criteria were as follows: (1) pathologically confirmed colon cancer and had undergone curative resection within 8 weeks; (2) stage III in TNM pathological staging according to the American Joint Committee on Cancer; (3) men or women aged from 18 to 75 years; (4) Karnofsky**’**s performance scoring (KPS) ≥70; (5) no strict heart, liver, kidney, or hematopoietic system disease or other factor affecting drug evaluation; and (6) volunteers who had given informed consent.

Patients with any of the following conditions were excluded: (1) mental disorder, pregnancy, or lactation; (2) participant in other clinical trial; (3) any adverse reaction to composition of PRM1201; and (4) incomplete information.

### Randomization and Blinding

Patients were randomly assigned to receive either chemotherapy plus PRM1201 (chemo+PRM1201) or chemotherapy plus placebo (chemo+placebo) at a ratio of 1:1. The randomization list was generated by SAS 8.2 and randomization was performed centrally by a statistician with no clinical involvement in the trial. PRM1201 and placebo were in granule form and identical in appearance, weight, color, smell, and taste. All physicians, researchers, and patients were blinded to treatment allocation.

### Intervention

PRM1201 is composed of seven herbs: *Ligustrum lucidum* W.T.Aiton fructus (Nv Zhen Zi), *Cistanche deserticola* Y.C.Ma herba (Rou Cong Rong), *Iphigenia indica* (L.) A.Gray ex Kunth pseudobulb (San Ci Gu), *Vitis quinquangularis* Rehder vine (Ye Pu Tao Teng), *Panax ginseng* C.A.Mey. root (Ren Sen), *Akebia trifoliata* (Thunb.) Koidz. fruit (Yu Zhi Zi), and *Salvia miltiorrhiza* Bunge root (Dan Shen). RPM1201 or placebo were administered twice a day from the start of adjuvant chemotherapy for a total of 6 months. PRM1201 (batch number: 1811334) were manufactured by Jiangyin Tian Jiang Pharmaceutical Co., Ltd. (Jiangsu Province, China). UPLC-Q-TOF/MS was used to analyze the chemical composition of PRM1201 (**Supplementary Table 1**). The details of total ion chromatogram of PRM1201 are shown in **Supplementary Figure 1**. PRM1201 was standardized to 12.0** g** per package and the color, shape, weight, smell, taste, and packaging of the placebo were similar to PRM1201. The intervention period lasted the entire chemotherapy cycles, 6 months in total. In addition, quality control of PRM1201 were evaluated by profiling of major phytochemical components at three time points (January 1 2016, May 7 2016, and October 12 2016). As shown in **Supplementary Figure 2**, both the overall cluster patterns and the percentages of individual major components indicated a good stability of PRM1201 used in the trial period.

All eligible patients were scheduled to receive standard adjuvant chemotherapy. The mFOLFOX6 or CapeOx regimen was recommended by the physician according to the National Comprehensive Cancer Network (NCCN) guidelines. Chemotherapy treatment was started within 8 weeks after surgery. Interruption happened when disease recurrence or metastasis was observed for any other reasons causing chemotherapy termination.

### Assessments

The primary endpoint was disease-free survival (DFS). This was calculated from the time of the intervention until any signs or symptoms of first recurrence or metastasis, or death from any cause be recorded. Patients were assessed before randomization. The baseline assessment involved a history and physical examination, complete blood count (CBC), liver and kidney function tests, carcinoembryonic antigen level measurement, chest computed tomography, and abdominal ultrasonography or computed tomography. Follow-up assessment consisted of periodic history and physical examination, adverse events, CBC, laboratory tests, and imaging studies. Colonoscopy was also performed yearly to assess recurrence and metastasis. Only imaging, cytologic analysis, or biopsy was accepted as evidence of recurrence or metastasis. The diagnosis of relapse could not be made on the basis of elevated carcinoembryonic antigen level. The Common Terminology Criteria of the National Cancer Institute (NCI-CTC, version 4.0) was used to grade treatment-emergent adverse events.

QOL scores and adverse events of patients were observed as the secondary endpoints. The European Organization for Research and Treatment of Cancer (EORTC) Quality of Life Questionnaire (QLQ-C30) was used to assess QOL (12). The QLQ-C30 includes 30 items to evaluate functional scales (physical, role, cognitive, emotional, and social functioning), global health status, symptom scales (fatigue, nausea and vomiting, and pain), and six single symptoms (dyspnea, insomnia, loss of appetite, constipation, diarrhea, and financial impact). The score of each item ranges from 1 (not at all) to 4 (very much), and raw scores were transformed to a 0-to-100 scale linearly. Higher scores on the functional scales and global QOL indicate a better QOL, whereas lower scores on the symptom scales indicate fewer complaints. When the score changed by over 10, this was seen as a significant variation (13).

### Statistical Analysis

*PASS version* 15.0.5 *(NCSS LLC.*, USA*)* was used for the sample size calculation. The 3-year disease-free survival (DFS) rate of stage III colon cancer patients after surgery in control group was estimated to be 68% (14, 15). Chemotherapy plus PRM1201 increased DFS to 79% in the adjuvant treatment of colon cancer and using an α value of 5% and power (1-β) of 80%, we calculated that a sample size of 327 was required. In consideration of a 10% loss rate in follow-up, there were a total of 364 cases needed in this study.

SPSS version 21.0 (IBM Corp., Armonk, USA) was used for the statistical analysis. DFS was compared using a two-sided log-rank test. Hazard ratios with 95% confidence intervals were calculated using the Cox proportional hazards model. The Kaplan–Meier method was used to draw survival curves. Standard parametric or non-parametric tests were used for continuous factors and χ2 tests were used for categorical factors. Continuous variables were presented as means (SD) and medians (interquartile range [IQR]). A value of *P* < 0.05 was considered significant.

## Results

### Patients’ Characteristics

**Figure 1:**
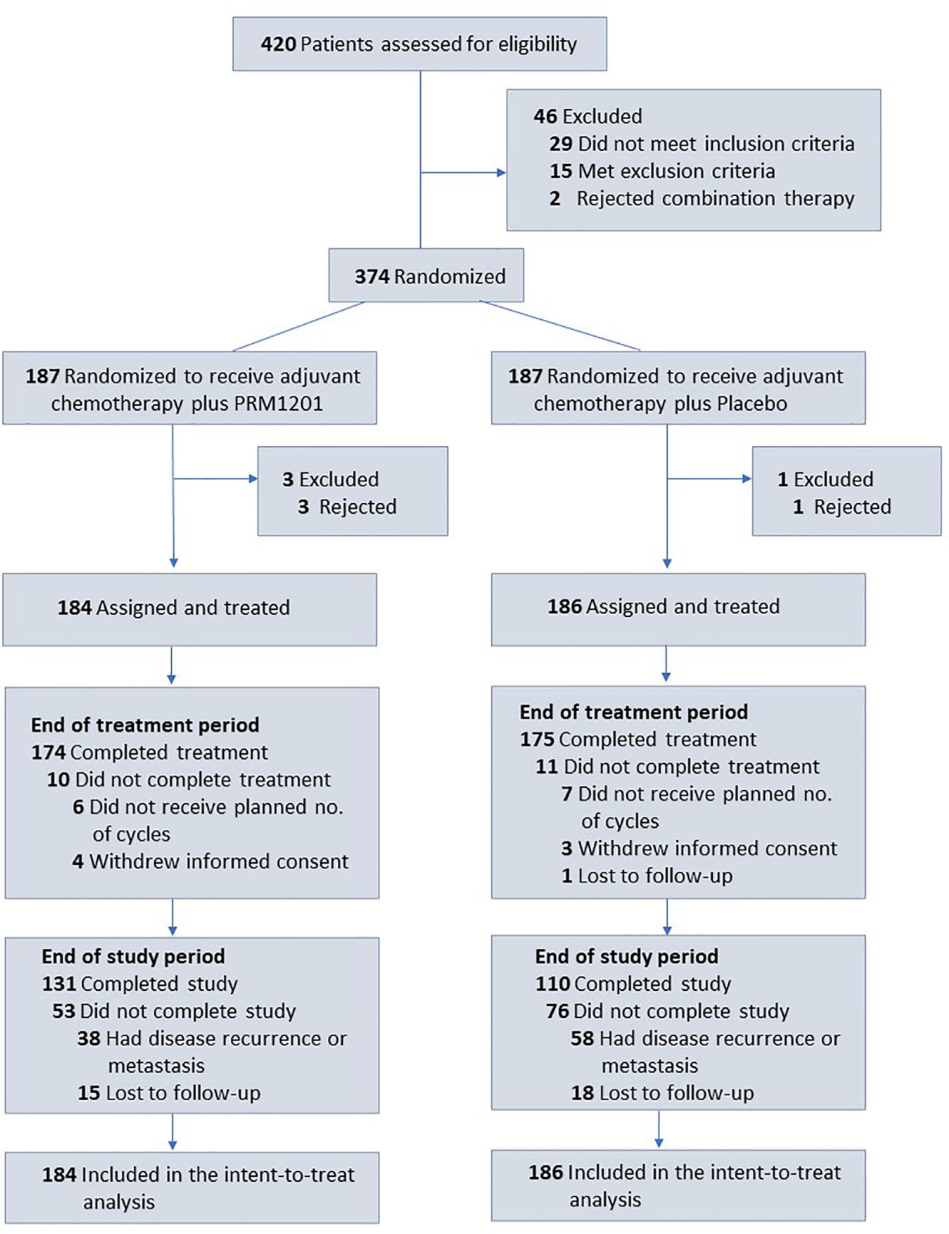
Flow of patients with colorectal cancer receiving adjuvant chemotherapy plus PRM1201 or Placebo.

Between January 2016 and June 2016, a total of 420 patients were screened from seven medical centers and 370 patients were included in the study. Patients were randomly assigned to receive chemotherapy plus PRM1201 (184 patients) or chemotherapy plus placebo (186 patients) (**Figure 1**). All patients received at least one cycle of treatment. The patients’ demographic and clinical characteristics were well matched between these two arms (**Table 1**). In both groups, the time between surgery and the start of chemotherapy were within 8 weeks.

**Table 1:** Characteristics of patients with colorectal cancer (n [%] or mean [SD]).

Characteristic	Chemotherapy+PRM1201	Chemotherapy+Placebo
Age (yr)		
Median	60.23 (8.7)	60.42 (9.3)
Range	33–75	32–75
Sex		
Male	105 (57.1)	107 (57.5)
Female	79 (42.9)	79 (42.5)
Karnofsky performance status (KPS) score		
70–80	28 (15.2)	24 (12.9)
80–100	156 (84.8)	162 (87.1)
Tumor stage		
T1-2	15 (8.1)	13 (7.0)
T3	104 (56.5)	104 (55.9)
T4	65 (35.3)	69 (37.1)
Nodal stage		
N1	116 (63.0)	114 (61.3)
N2	68 (37.0)	72 (38.7)
Histologic appearance		
Well differentiated	7 (3.8)	7 (3.8)
Moderate differentiated	146 (79.3)	151 (81.2)
Poor differentiated	31 (16.9)	28 (15.0)
Chemotherapy regimen		
CapeOx	82 (44.6)	85 (46.2)
FOLFOX	102 (55.4)	100 (53.8)

### Disease-Free Survival

**Figure 2:**
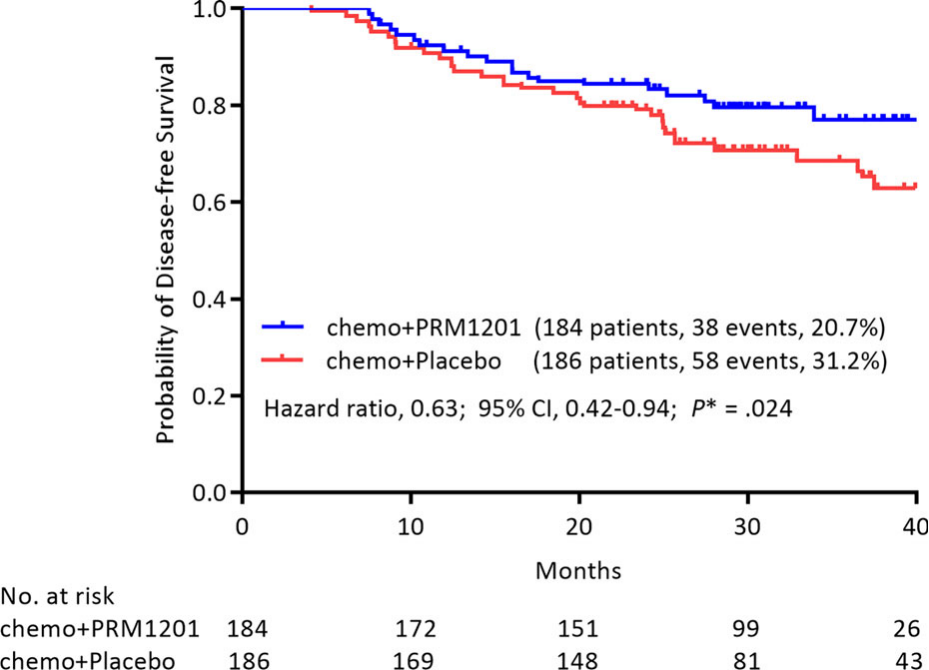
Kaplan- Meier estimates of DFS in the group given chemotherapy plus PRM1201 and the group given chemotherapy plus placebo, according to the intention to treat.

At the time of analysis (median follow-up, 29.60 months), 38 (20.4%) patients in the group given chemotherapy plus PRM1201 had relapsed or died, as compared with 58 (31.5%) in the control group (**Table 2**). The 1-year, 2-year, and 3-year DFS rates were 91.2, 84.5, and 77.1% in the chemo+PRM1201 arm, and they were 89.7, 79.2, and 68.6% in the chemo+Placebo arm respectively, with a *P* value of 0.024. This result indicated that the DFS in the group given chemotherapy plus PRM1201 was significantly higher than in the control group. The hazard ratio (HR) for recurrence in the group given chemotherapy plus PRM1201, as compared with the control group was 0.63 (95% Confidence Interval [CI], 0.42 to 0.94), corresponding to a 37% reduction in the risk of recurrence (**Figure 2**).

**Table 2:** Analysis of disease-free survival according to the intention-to-treat population (n [%] or %).

Variable	Chemo+PRM1201 (n = 184)	Chemo+Placebo (n = 186)
3-yr DFS rate*	77.1%	68.6%
Event free	146 (79.3)	128 (68.8)
Any DFS event		
Metastasis	31 (16.8)	47 (25.3)
Local recurrence	5 (2.7)	8 (4.3)
Death	2 (1.1)	3 (1.6)

When age (≥65y, or <65y), sex, tumor stage (T1-2, T3, or T4), and nodal stage (N1, or N2) were included in the analysis using a Cox model with the treatment for the DFS end point, T4 tumor stage (HR, 2.874; 95% CI, 1.035 to 7.986, *P* = 0.043), and N2 nodal stage (HR, 2.709; 95% CI, 1.790 to 4.099, *P* < 0.001) remained in the model (**Table 3**). Among all variables listed above, the HR for PRM1201 *versus* placebo was 0.636 (95% CI, 0.421 to 0.960; *P* = 0.031).

**Table 3:** Multivariate cox proportional hazards regression analysis of DFS, according to the intention to treat.

Prognostic Factor	Hazard Ratio	95% CI	*P* value
PRM1201 therapy	0.636	0.421–0.960	0.031
Age ≥65 yr	0.950	0.624–1.444	0.809
Female sex	1.029	0.682–1.553	0.892
T3 tumor stage	1.632	0.581–4.584	0.353
T4 tumor stage	2.874	1.035–7.986	0.043
N2 nodal stage	2.709	1.790–4.099	<0.001

### Quality of Life

**Figure 3:**
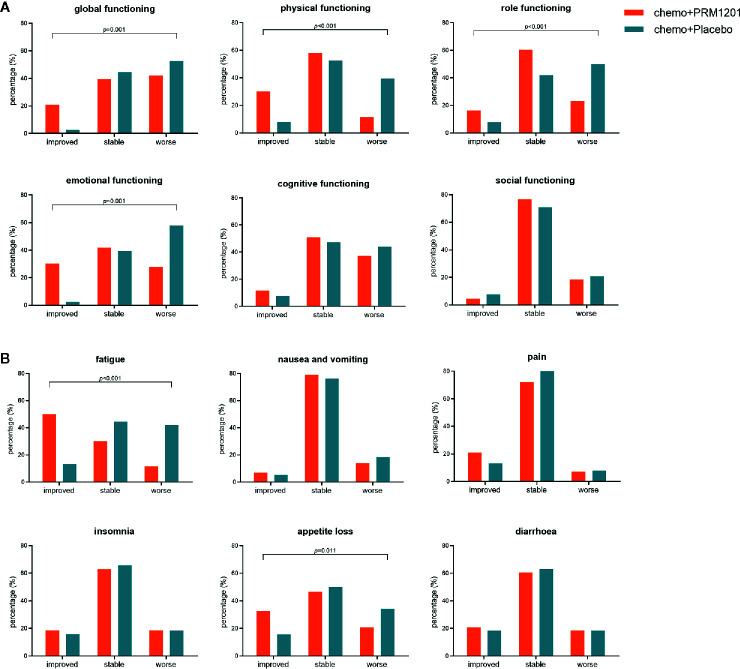
Proportion of patients with improved, stable, worsened quality of life in several domains and symptoms after treatment according to QLQ-C30. **(A)** for global health status and five functional scales (physical, role, cognitive, emotional, and social functioning); **(B)** for three symptom scales (fatigue, nausea and vomiting, and pain) and three single symptoms with high scores (insomnia, appetite loss, and diarrhea).

EORTC QLQ-C30 scores were used to assess the QOL of patients. Patients were evaluated using the questionnaire for the baseline QOL assessment, and there was no significant difference in baseline scores in all domains and items between these two groups (**Supplementary Figure 3**). The most common symptoms were fatigue (mean 31.41 ± 15.69) for CRC patients after curative resection, followed by appetite loss (mean 23.51 ± 22.86) and insomnia (mean 16.70 ± 19.49). For functioning scales, the improvement ratios in the global functioning (10.9 *vs.* 2.6%), physical functioning (30.2 *vs.* 7.9%), role functioning (16.3 *vs.* 7.8%), emotional functioning (30.2 *vs.* 2.6%) were significantly higher in the group receiving chemotherapy plus PRM1201 than in the chemotherapy plus placebo group (*P* <0.05) (**Figure 3A**). For symptom scales, the improvement ratios in fatigue (50.1 *vs.* 13.2%), and appetite loss (32.6 *vs.* 15.8%) were significantly higher in the group given chemotherapy plus PRM1201 than in the control group (*P* < 0.05) (**Figure 3B**).

### Adverse Events

There was no serious adverse event that occurred in either group. Neutropenia (108 of 370 patients), diarrhea (43 of 370 patients), and paresthesia (42 of 370 patients) were the most grade 3 or 4 treatment- related adverse events (TRAEs) in all patients with adjuvant treatment. Grade 3 or 4 neutropenia was less common in patients with chemotherapy plus PRM1201 than those with chemotherapy plus placebo (*P* = 0.292). The proportions of patients with chemotherapy related diarrhea (*P* = 0.545), vomiting (*P* = 0.371), and nausea (*P* = 0.317) were also lower in the PRM1201 arm than in the control arm. While the incidence of other grade 3 or 4 TRAEs between both arms was similar (**Table 4**).

**Table 4:** Adverse events in the group given chemotherapy plus PRM1201 and chemotherapy plus Placebo (n [%]).

Adverse Event	Chemo+PRM1201		Chemo+Placebo		*P* value
	Grade 3	Grade 4	Grade 3	Grade 4	
Neutropenia	36 (19.6)	11 (6.0)	45 (24.5)	16 (8.6)	0.292
Anemia	3 (1.6)	2 (1.1)	4 (2.2)	2 (1.1)	0.935
Thrombocytopenia	4 (2.2)	2 (1.1)	6 (3.2)	2 (1.1)	0.609
Nausea^†^	6 (3.2)	NA	10 (5.5)	NA	0.317
Diarrhea	16 (8.7)	2 (1.1)	22 (11.8)	3 (1.6)	0.545
Vomiting	8 (4.3)	2 (1.1)	14 (7.5)	1 (0.1)	0.371
Paresthesia^†^	22 (12.0)	NA	20 (10.8)	NA	0.715
Hand-foot Syndrome	4 (2.2)	1 (0.1)	4 (2.2)	2 (1.1)	0.850

^†^There are only three grades of nausea and paresthesia in the Common Terminology Criteria of the National Cancer Institute (NCI-CTC, version 4.0). NA demotes not applicable.

## Discussion

Resection and chemotherapy are the most effective therapies in clinical management for stage III colon cancer patients (16–18). Oxaliplatin (19) and capecitabine (20) are the most commonly used chemotherapeutic drugs, and FOLFOX and CapeOx regimens are the first-line chemotherapy for stage III colon cancer according to the NCCN guidelines. A high-quality meta-analysis showed that the 5-year DFS rates of stage II and III colorectal cancer patients receiving assisted chemotherapy were 79.3 and 63.6%, respectively (21). The MOSAIC study, which had a 9.5-year follow-up period, showed that the 5-year DFS rate of stage III CRC patients in the FOLFOX group was 66.4% (22). There is a consensus that postoperative recurrence and metastasis mainly occur in the first 2 years after surgery and that improving the 2-year DFS should be the main focus of preventing disease progression. In our study, the 1-year, 2-year, and 3-year rates of DFS were 89.7, 79.2, and 68.6% in patients receiving chemotherapy plus placebo, similar to 69.3% and 60.68% to 69.15% reported by the other two clinical trials in China (14, 15).

In China, CHM combined with chemotherapy is commonly used in clinical practice. A number of clinical studies have confirmed that CHM can reduce the toxicity of chemotherapeutic drugs, but the effects of CHM on survival outcomes remain undefined. In this study, our primary outcome was DFS. It was shown that the 1-year, 2-year, and 3-year rates of DFS were 91.2, 84.5, and 77.1% in the group given chemotherapy plus PRM1201. It suggested that the herbal medicine PRM1201 can reduce recurrence and metastasis and can prolong the DFS of patients with stage III colon cancer.

Although chemotherapy can significantly prolong DFS and OS, it causes a lot of negative effects, such as neutropenia, dehydration (23), and peripheral sensory neuropathy (24). It was reported that 12.4% of oxaliplatin patients suffered grade 3 peripheral neurotoxicity (25), and 20–50% of them had to reduce their dosage or even stop taking treatment because of intolerable drug related toxicity (26), leading to worse outcomes (21). According to Baek Y**’**s research, lack of appetite, insomnia, and fatigue are the most frequently observed symptoms in patients receiving chemotherapy in South Korea (27), and all of these symptoms can significantly reduce the QOL of patients (28). Many clinical studies have demonstrated a reduction in the QLQ of colorectal cancer patients in western countries (29–31). In our study, all of these adverse effects and the reduction of QLQ-C30 scores were observed in patients receiving chemotherapy plus placebo.

Therefore, CHM combinational therapy has a distinct advantage in the treatment of cancers. It is generally acknowledged in China, and in Asia as a whole, that CHM can promote the quality of life, alleviate the adverse effects of chemotherapy, improve the effects of chemotherapy, and relieve negative clinical symptoms of cancer patients. The PRM1201 can significantly reverse the decline in QLQ, especially in terms of improved scores in the global functioning, physical functioning, role functioning, emotional functioning, fatigue, and appetite loss. We also observed the decreases in these adverse effects and QLQ-C30 scores in patients who received the placebo. At the same time, the effects of reducing the incidence of grade 3 or 4 neutropenia of PRM1201 were observed. However, the adverse effects mentioned above are not the main outcome of this study. The evaluation of this part has certain limitations, and further observations and improved efficacy evaluation are needed. During the trial period, there were no major side effects of RPM1201 other than few reports of increased frequency of diarrhea. While the diarrhea can produce tolerance in short time after persistent receiving PRM1201 and there were no differences between two groups in diarrhea according to QOL score or TRAEs.

The clinical efficacy of CHM in preventing recurrence and metastasis of colon cancer is attributed to its multiple pharmacological activities, such as inhibiting angiogenesis, reducing chemotherapeutic drug resistance, regulating immunity, and improving the tumor microenvironment. In our preliminary study, we detected serum VEGF, MMP-2, and MMP-9 in patients with colon cancer. It was found that PRM1201 can inhibit the expression of angiogenetic factors, indicating that the effect of RPM1201 on anti-tumor metastasis might be related to the inhibition of angiogenesis. In combination with basic experimental studies, the mechanism of anti-metastasis of PRM1201 is associated with the regulation of Wnt/β-catenin signaling pathway and HIPK2-p53 signaling pathways (9, 10). Through clinical observations, it was found that the clinical efficacy of PRM1201 might be associated with the regulation of intestinal microbiota. Further rigorously designed study is needed to confirm this assumption.

This study was a perspective, randomized, double-blinded, placebo-controlled clinical trial. We focused on the effect of PRM1201 in preventing the recurrence and metastasis of stage III colon cancer treated with chemotherapy after surgery. We confirmed that PRM1201 can prolong the DFS of patients with stage III colon cancer, and improve the QOL of patients. However, the anti-tumor mechanism of the THM formula is not fully understood, and further studies are warranted.

## Conclusions

This clinical trial demonstrated that oral PRM1201 significantly improve the DFS of patients with stage III colon cancer with adjuvant chemotherapy after surgery. It showed that PRM1201 has great potential to increase survival outcome, prevent recurrence and metastasis, and improve the QOL of patients. PRM1201 can be recommended as an effective option in clinical practice.

## Data Availability Statement

The original contributions presented in the study are included in the article/**Supplementary Material**. Further inquiries can be directed to the corresponding authors.

## Ethics Statement

The studies involving human participants were reviewed and approved by IRB of Shuguang Hospital affiliated with Shanghai University of TCM. The patients/participants provided their written informed consent to participate in this study.

## Author Contributions

QL was involved in the conception and design of the study. RJ, NL, GC, HX, PZ, HML, JH, XY, HG, WY and QL were involved in provision of study materials or patients. RJ, YuZ, LHZ, YiZ, CW, XS, YF, HJL, HL, ZH, YL, YaZ and GH were involved in acquisition of data. RJ, QJ, ZL and WY were involved in data analysis and interpretation. RJ drafted the manuscript and QL, WY and NL were involved in the critical revision of the manuscript. All authors contributed to the article and approved the submitted version.

## Funding

This research was supported by grants from the Key projects of the National Natural Science Foundation of China (No.82030118), Three-year Plan of Action for the Development of Traditional Chinese Medicine in Shanghai (ZY(2018-2020)-CCCX-2003-03), and key Projects of Shanghai Science and Technology Commission (16401970500).

## Conflict of Interest

The authors declare that the research was conducted in the absence of any commercial or financial relationships that could be construed as a potential conflict of interest.
