# Supplementary material for: Effect of PRM1201 Combined With Adjuvant Chemotherapy on Preventing Recurrence and Metastasis of Stage III Colon Cancer: A Randomized, Double-Blind, Placebo-Controlled Clinical Trial
Source: Front Oncol. 2021 Mar 3;11:618793. doi: 10.3389/fonc.2021.618793 (PMC7968418; doi:10.3389/fonc.2021.618793)
Supplement: Supplementary file 1 [file Table_1.docx]

A.

B.

Suppl. Figure 1 UPLC-HRMS total ion chromatogram of PRM1201

(A. for Negative ion mode and B. for positive ion mode.)

Suppl. Table 1 exact chemical composition of PRM1201

| No. | Retention time  (min) | ppm | Formula | Name | MS/MS spectra |
| --- | --- | --- | --- | --- | --- |
| 1 | 0.85 | 3.1 | C_7_H_12_O_6_ | Quinic acid | 191.0568;127.0411;93.0350;87.0091;85.0301;59.0142 |
| 2 | 3.06 | 2.8 | C_9_H_10_O_5_ | Danshensu | 179.0366;135.0459;123.0460;72.9936 |
| 3 | 3.67 | 3.4 | C_16_H_22_O_10_ | Geniposidic acid | 373.1152;211.0593;149.0612;123.0452 |
| 4 | 5.08 | 4.9 | C_16_H_24_O_10_ | 8-epi-loganic acid | 375.1315;331.1439;213.0770;151.0766 |
| 5 | 5.42 | 2.4 | C_26_H_40_O_17_ | Kankanoside F | 623.2205;461.1670 |
| 6 | 5.76 | 6.4 | C_7_H_6_O_3_ | p-Hydroxybenzoic acid | 137.0244;108.0215;81.0345;65.0027 |
| 7 | 5.8 | 5.7 | C_16_H_24_O_10_ | Loganic acid | 375.1315;331.1439;151.0766;125.0634 |
| 8 | 6.17 | -1.8 | C_17_H_22_O_13_ | / | 433.0962;389.1172;209.0454;177.0189 |
| 9 | 8.57 | 1.2 | C_21_H_28_O_13_ | Cistanoside F | 487.1387;323.0773;251.0561;179.0350;161.0248;135.0451 |
| 10 | 10.77 | -3.6 | C_35_H_46_O_21_ | Cistantubuloside | 801.2467;783.2328;621.2078;179.0368;161.0256 |
| 11 | 13.14 | 1.4 | C_35_H_46_O_20_ | Echinacoside | 785.2484;623.2178;161.0249 |
| 12 | 14.17 | -1 | C_20_H_22_O_8_ | Reseratroloside | 227.0689 |
| 13 | 14.64 | -0.7 | C_35_H_46_O_19_ | Poliumoside | 769.2531;623.2575;161.0238 |
| 14 | 15.28 | -0.5 | C_31_H_42_O_18_ | Neonuzhenide | 701.2230;539.1797;469.1304;315.1050 |
| 15 | 16.13 | 3.3 | C_29_H_36_O_15_ | Acteoside | 623.1974;461.1663;161.0249 |
| 16 | 16.19 | 0 | C_37_H_48_O_21_ | Tubuloside A | 827.2617;665.2270;623.2154;161.0244 |
| 17 | 17.1 | -0.5 | C_31_H_42_O_17_ | Specnuezhenide | 685.2404;523.1846;453.1431;421.1528;299.1143 |
| 18 | 17.14 | 2.2 | C_29_H_36_O_15_ | Isoacteoside | 623.2001;461.1661;161.0258 |
| 19 | 17.32 | 1.1 | C_20_H_18_O_10_ | Salvianolic acid D | 417.0841;373.0923;197.0464;175.0404 |
| 20 | 17.91 | 0.8 | C_31_H_42_O_17_ | Isonuezhenide | 685.2368;523.1875;453.1429;421.1519;299.1125;223.0639 |
| 21 | 18.72 | 0.3 | C_36_H_30_O_16_ | Salvianolic acid E | 717.1496;519.0939;339.0505;321.0395 |
| 22 | 19.85 | 1.6 | C_27_H_22_O_12_ | Lithospermic acid | 493.1124;295.0603;185.0241 |
| 23 | 19.85 | -2.5 | C_14_H_12_O_3_ | Resveratrol | 185.0609;159.0835 |
| 24 | 21.77 | 1.8 | C_36_H_30_O_16_ | Salvianolic acid B | 717.1467;519.0934;339.0511;321.0405 |
| 25 | 23.06 | -0.2 | C_36_H_30_O_16_ | Salvianolic acid L | 717.1407;519.0934;339.0502;321.0395 |
| 26 | 24.12 | 1.4 | C_36_H_30_O_16_ | Salvianolic acid Y | 717.1545;519.0938;339.0545;321.0410 |
| 27 | 24.31 | 2.2 | C_26_H_22_O_10_ | Salvianolic acid A | 295.0620;185.0262 |
| 28 | 25.46 | -0.4 | C_48_H_64_O_27_ | G13 | 1071.3576;771.2452;685.2334;523.1785;453.1380 |
| 29 | 27.18 | 3.4 | C_48_H_64_O_27_ | G13 isomer | 1071.3635;909.3132;839.2505;685.2417;453.1388 |
| 30 | 31.35 | 5.5 | C_59_H_96_O_26_ | Saponins PK | 749.4476;469.1533 |
| 31 | 32.99 | -0.7 | C_52_H_84_O_22_ | Saponins E | 1059.5476;735.4368;603.4024;323.1076 |
| 32 | 33.35 | 2.8 | C_53_H_86_O_22_ | Saponins PJ-2 | 1073.5602;749.4530;323.0985 |
| 33 | 34.22 | 4.3 | C_47_H_76_O_18_ | Saponins D | 927.5262;603.3769;323.0964 |
| 34 | 38.8 | 3.8 | C_53_H_90_O_22_ | Ginsenoside Rb2 | 1123.5895;1077.5863;915.5316;783.4868 |
| 35 | 39.03 | 4.9 | C_53_H_90_O_22_ | Ginsenoside Rb3 | 1123.5957;1077.5882;945.5386;915.5394;783.4958;621.4545 |
| 36 | 39.15 | 3.1 | C_52_H_84_O_21_ | Yuzhizioside Ⅳ | 1043.5445;719.4398;323.1008 |
| 37 | 39.38 | 3.5 | C_48_H_82_O_18_ | Gypenoside XVII | 991.5505;945.5430;783.4920;621.4401 |
| 38 | 40.17 | 0.7 | C_47_H_76_O_17_ | Pulsatilla Saponins D | 911.4975;749.4455;603.3937 |
| 39 | 40.26 | 4.6 | C_42_H_70_O_12_ | Ginsenoside F4 | 811.4934;765.4843;619.4287 |
| 40 | 40.5 | 1.4 | C_40_H_64_O_12_ | Saponins B | 735.4339;603.3899;471.3468 |
| 41 | 40.59 | -0.6 | C_41_H_66_O_12_ | Saponins Pd | 749.4535;703.4466;603.3849 |
| 42 | 40.72 | 5.7 | C_42_H_72_O_13_ | Ginsenoside Rg3 | 783.4951;621.4429;161.0465;101.0264 |
| 43 | 40.97 | 2.2 | C_35_H_56_O_8_ | Saponins A | 603.3904;325.1053 |
| 44 | 41.24 | -2.1 | C_19_H_20_O_3_ | Isocryptotanshinone | 297.1466;253.1567;237.0894;209.0958 |
| 45 | 41.7 | -2 | C_18_H_14_O_3_ | Dihydrotanshinone I | 279.1007;261.0898;233.0952;205.1008 |
| 46 | 41.8 | 1.1 | C_42_H_70_O_12_ | Ginsenoside Rk1 | 811.4920;765.4856;603.4334 |
| 47 | 42.07 | -2.4 | C_20_H_18_O_5_ | Methyl tanshinate | 279.1012;261.0909;233.0963;205.1010 |
| 48 | 42.61 | -0.8 | C_18_H_12_O_3_ | Tanshinone I | 277.0861;249.0909;234.0672;221.0961 |
| 49 | 42.71 | -0.7 | C_19_H_20_O_3_ | Cryptotanshinone | 297.1473;268.1079;251.1422;237.0905 |
| 50 | 43.48 | -2.3 | C_19_H_18_O_3_ | Tanshinone IIA | 295.1310;280.1076;277.1205;249.1261 |


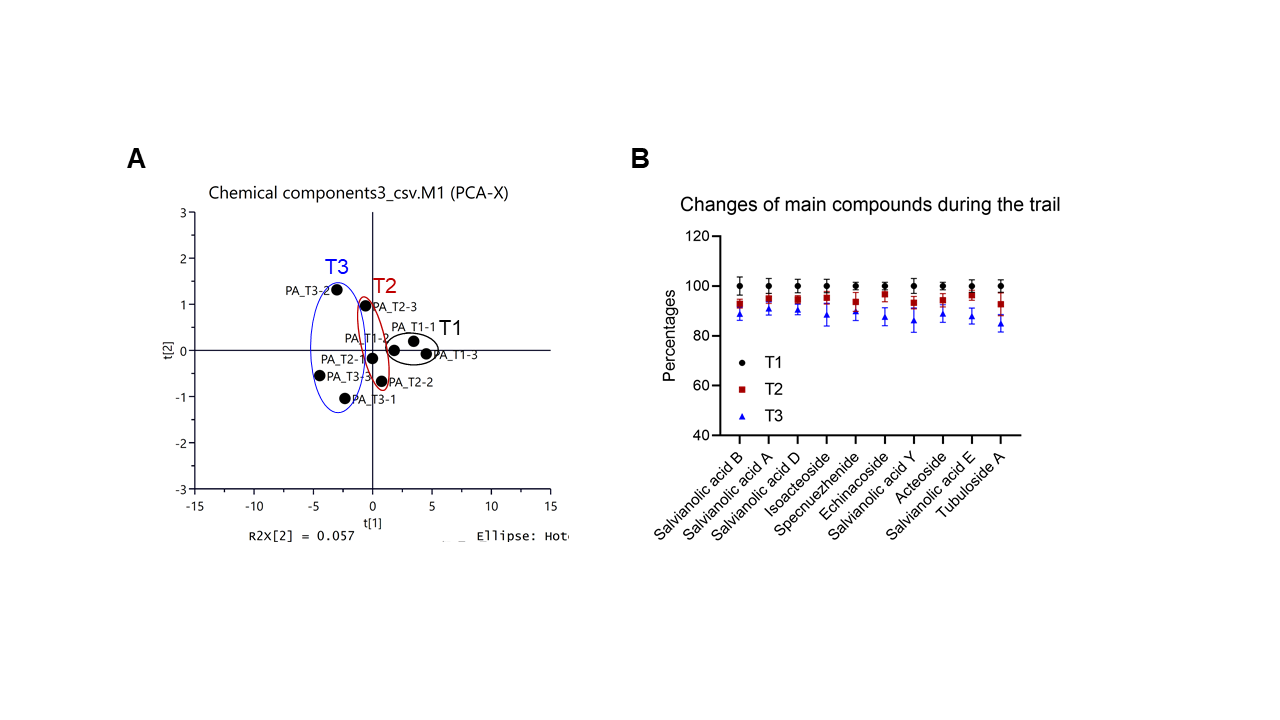


Suppl. Figure 2 Profiling of major phytochemical components at three time points of PRM1201


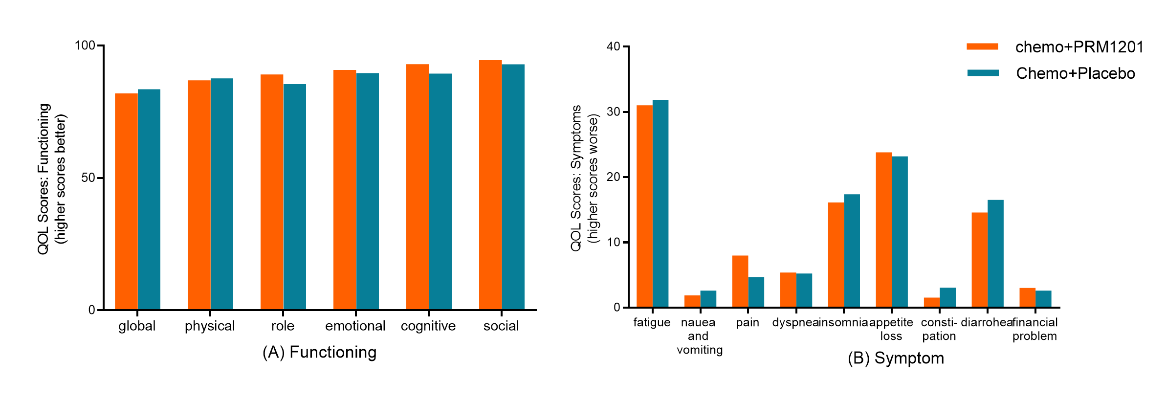


Suppl. Figure 3. Baseline of quality-of-life (QOL)-C30 scores in the two arms. (A) for functional scales (physical, role, cognitive, emotional, and social functioning), global health status; (B) for symptom scales (fatigue, nausea and vomiting, and pain) and six single symptoms (dyspnea, insomnia, loss of appetite, constipation, diarrhea, and financial impact)
